# Supplementary material for: Smartphone ownership, digital literacy, and the mediating role of social connectedness and loneliness in improving the wellbeing of community-dwelling older adults of low socio-economic status in Singapore
Source: PLoS One. 2023 Aug 30;18(8):e0290557. doi: 10.1371/journal.pone.0290557 (PMC10468072; doi:10.1371/journal.pone.0290557)
Supplement: S1 Appendix — (DOCX) [file pone.0290557.s001.docx]

## Supplementary Materials

Table S1. Digital Literacy Survey, and Rating and Coding Scheme

| **Aspects of Literacy** | **Smart-phone Functions** | **Utilization Coding Scheme^a^** |
| --- | --- | --- |
| Sociability | Text Message | 0 = F; 1 = A to E |
|  | Video Call | 0 = F; 1 = A to E |
|  | Voice Call | 0 = F; 1 = A to E |
| Instrumental | Government Apps | 0 = F; 1 = A to E |
|  | Online Banking | 0 = F; 1 = A to E |
|  | Online Purchase | 0 = F; 1 = A to E |
|  | Health Apps | 0 = F; 1 = A to E |
|  | Read News | 0 = F; 1 = A to E |
| Reassurance | Call Ambulance | 0 = F; 1 = A to E |
|  | Call Police | 0 = F; 1 = A to E |
| Pastime | Listen to Music | 0 = F; 1 = A to E |
|  | Watch Video | 0 = F; 1 = A to E |
|  | Play Games | 0 = F; 1 = A to E |
| ^a^ The participants were asked to rate each of the functions on a 7-point A to F scale on their proficient and frequency of usage, where A = I know how to use it and I use it every day; B = I know how to use it and I use it 5-6x/week; C = I know how to use it and I use it 3-4x/week; D = I know how to use it and I use it 1-2x/week; E = I know how to use it and I use it < 1x/week; F = I do not know how to use it. These were recoded to 0 = No utilization and 1 = Utilized for further analyses. | | |

Table S2. Descriptive statistics of key variables

| **Variables** | | | **Theoretical Minimum and Maximum** | **Median (IQR)^a^** | **% Utilized^b^** |
| --- | --- | --- | --- | --- | --- |
| **Overall Digital Literacy Index** | | | 0 – 13 | 4 (5) |  |
|  | Sociability Literacy Index | | 0 – 3 | 2 (2) |  |
|  |  | Text Message | 0 – 1 |  | 37.36 |
|  |  | Video Call | 0 – 1 |  | 32.59 |
|  |  | Voice Call | 0 – 1 |  | 73.43 |
| **Instrumental Literacy Index** | | | 0 – 5 | 0 (1) |  |
|  | Government Apps | | 0 – 1 |  | 10.00 |
|  | Online Banking | | 0 – 1 |  | 7.78 |
|  | Online Purchase | | 0 – 1 |  | 7.18 |
|  | Health Apps | | 0 – 1 |  | 11.52 |
|  | Read News | | 0 – 1 |  | 30.74 |
| **Reassurance Literacy Index** | | | 0 – 2 | 2 (2) |  |
|  | Call Ambulance | | 0 – 1 |  | 50.56 |
|  | Call Police | | 0 – 1 |  | 50.19 |
| **Pastime Literacy Index** | | | 0 – 3 | 1 (2) |  |
|  | Listen to Music | | 0 – 1 |  | 34.94 |
|  | Watch Video | | 0 – 1 |  | 40.81 |
|  | Play Games | | 0 – 1 |  | 25.37 |
| **UCLA-3 Loneliness** | | | 3 – 9 | 3 (1) |  |
| **LSNS-6 Connectedness** | | | 0 – 30 | 10 (9) |  |
| **Personal Wellbeing Scale** | | | 0 – 12 | 8 (4) |  |
| **EQ-5D-3L Utility Score** | | | -0.769 – 1 | 0.854 (0.314) |  |
| **EQ VAS** | | | 0 – 100 | 70 (30) |  |
| ^a^ *IQR =* Inter-quartile range  ^b^ % Utilized = percentages of the samples who reported utilizing the function. | | | | | |

Table S3. Factors associated with digital literacy.

|  |  | **Digital Literacy Index^a^** | |  | **Univariable Tests** |  | **Multivariable Logistic Regression^b^** | |
| --- | --- | --- | --- | --- | --- | --- | --- | --- |
| **Variables** | | **Low (n = 141)** | **High (n = 126)** |  | **P-value^c^** |  | **Odds Ratio [95%CI]** | **P-value** |
| **Age (year), Median (IQR)** | | 76 (15) | 69.5 (11) |  | <.001** |  | 0.99 [0.98, 1.00] | .048 |
| **Gender, n (%)** | |  |  |  |  |  |  |  |
|  | Female | 99 (65.1) | 53 (34.9) |  | <.001* |  | 0.49 [0.28, 0.85] | .011 |
|  | Male | 42 (36.5) | 73 (63.5) |  |  |  | -^d^ | - |
| **Race, n (%)** | |  |  |  |  |  |  |  |
|  | Chinese | 116 (56.3) | 90 (43.7) |  | 0.050* |  | - | - |
|  | Non-Chinese | 25 (41.0) | 36 (59.0) |  |  |  | - | - |
| **Housing Type, n (%)** | |  |  |  |  |  |  |  |
|  | Rented | 116 (52.7) | 104 (47.3) |  | 0.661* |  | - | - |
|  | Owned | 20 (47.6) | 22 (52.4) |  |  |  | - | - |
| **Apartment Size, n (%)** | |  |  |  |  |  |  |  |
|  | One room | 80 (54.1) | 68 (45.9) |  | 0.866* |  | - | - |
|  | Two room | 47 (50.5) | 46 (49.5) |  |  |  | - | - |
|  | Larger than Two room | 13 (52.0) | 12 (48.0) |  |  |  | - | - |
| **Education Level, n (%)** | |  |  |  |  |  |  |  |
|  | No formal education | 69 (72.6) | 26 (27.4) |  | <.001* |  | -^d^ | - |
|  | Primary education | 55 (50.5) | 54 (49.5) |  |  |  | 2.47 [1.36, 4.49] | .003 |
|  | Secondary and Tertiary education | 17 (28.3) | 41 (71.7) |  |  |  | 6.17 [2.98, 12.74] | < .001 |

^a^ Grouping of low and high digital literacy was done using a median-split at the value of 3.

^b^ Only variables with statistically significant association found in the univariable tests were included in the logistic regression model. After listwise exclusion of those with missing data, 259 participants were included in the final regression model.

^c^ *Chi-squared Test. **Mann-Whitney U-Test.

^d^ Reference group of the variable.

Table S4. Factors associated with smartphone ownership.

|  | | **Smartphone ownership** | |  | **Univariable Tests** |  | **Multivariable Logistic Regression^a^** | |
| --- | --- | --- | --- | --- | --- | --- | --- | --- |
| **Variables** | | **No (n = 121)** | **Yes (n = 181)** |  | **P-value^b^** |  | **Odds Ratio [95% CI]** | **P-value** |
| **Age (year), Median (IQR)** | | 76 (15) | 70 (10) |  | <.001** |  | 0.99 [0.90, 1.00] | .044 |
| **Gender, n (%)** | |  |  |  |  |  |  |  |
|  | Female | 74 (44.3) | 93 (55.7) |  | .120* |  | - | - |
|  | Male | 47 (34.8) | 88 (65.2) |  |  |  | - | - |
| **Race, n (%)** | |  |  |  |  |  |  |  |
|  | Chinese | 100 (42.7) | 134 (57.3) |  | .106* |  | - | - |
|  | Non-Chinese | 21 (30.9) | 47 (69.1) |  |  |  | - | - |
| **Education Level, n (%)** | |  |  |  |  |  |  |  |
|  | No formal education | 57 (55.8) | 45 (44.1) |  | <.001* |  | -^^^ | - |
|  | Primary Education | 48 (38.1) | 78 (61.9) |  |  |  | 2.37 [1.42, 3.98] | .001 |
|  | Secondary and Tertiary education | 15 (21.1) | 56 (78.9) |  |  |  | 5.41 [2.86, 10.62] | <.001 |
| **Housing Type, n (%)** | |  |  |  |  |  |  |  |
|  | Rented | 103 (40.9) | 149 (59.1) |  | .431* |  | - | - |
|  | Owned | 15 (33.3) | 30 (66.7) |  |  |  | - | - |
| **Apartment Size, n (%)** | |  |  |  |  |  |  |  |
|  | One room | 72 (42.1) | 99 (57.9) |  | .207* |  | - | - |
|  | Two room | 35 (33.7) | 69 (66.3) |  |  |  | - | - |
|  | Larger than Two room | 13 (50.0) | 13 (50.0) |  |  |  | - | - |

^a^ Only variables with statistically significant association found in the univariable tests were included in the logistic regression model. After listwise exclusion of those with missing data, 259 participants were included in the final regression model.

^b^ *Chi-squared Test. **Mann-Whitney U-Test.

^c^ Reference group of the variable.

Table S5. Results of Serial Mediation Model a, b

| **Total, Direct, and Indirect Effects** | | $\hat{\boldsymbol{\beta}}$ | **95% CI** | **P-value** | ${\hat{\boldsymbol{\beta}}}_{\boldsymbol{std}}$ |
| --- | --- | --- | --- | --- | --- |
| Total effect of Social Literacy on Wellbeing | | 0.648 | 0.111, 1.147 | .016 | 0.238 |
|  | Direct effect: Social **->** Wellbeing | 0.367 | -0.144, 0.816 | .133 | 0.135 |
|  | Indirect effect 1: Social **->** Connectedness **->** Wellbeing | 0.181 | 0.032, 0.393 | - | 0.066 |
|  | Indirect effect 2: Social **->** Loneliness **->** Wellbeing | 0.063 | -0.092, 0.239 | - | 0.023 |
|  | Indirect effect 3: Social **->** Connectedness **->** Loneliness **->** Wellbeing | 0.037 | 0.007, 0.101 | - | 0.013 |
| Total effect of Social Literacy on QoL (VAS) | | 2.369 | -1.030, 5.848 | .178 | 0.128 |
|  | Direct effect: Social **->** QoL (VAS) | 1.756 | -1.620, 5.198 | .313 | 0.095 |
|  | Indirect effect 1: Social **->** Connectedness **->** QoL (VAS) | 0.372 | -0.071, 1.317 | - | 0.020 |
|  | Indirect effect 2: Social **->** Loneliness **->** QoL (VAS) | 0.152 | -0.160, 0.888 | - | 0.008 |
|  | Indirect effect 3: Social **->** Connectedness **->** Loneliness **->** QoL (VAS) | 0.088 | -0.001, 0.339 | - | 0.005 |
| Total effect of Social Literacy on QoL (Utility) | | 0.043 | -0.013, 0.095 | .114 | 0.174 |
|  | Direct effect: Social **->** QoL (Utility) | 0.027 | -0.023, 0.075 | .270 | 0.111 |
|  | Indirect effect 1: Social **->** Connectedness **->** QoL (Utility) | 0.006 | 0.000, 0.018 | - | 0.025 |
|  | Indirect effect 2: Social **->** Loneliness **->** QoL (Utility) | 0.006 | -0.009, 0.023 | - | 0.024 |
|  | Indirect effect 3: Social **->** Connectedness **->** Loneliness **->** QoL (Utility) | 0.004 | 0.001, 0.010 | - | 0.014 |
| Total effect of Instrumentality Literacy on Wellbeing | | -0.113 | -0.552, 0.335 | .618 | -0.048 |
|  | Direct effect: Instrumentality **->** Wellbeing | 0.005 | -0.446, 0.417 | .981 | 0.002 |
|  | Indirect effect 1: Instrumentality **->** Connectedness **->** Wellbeing | -0.149 | -0.309, -0.034 | - | -0.063 |
|  | Indirect effect 2: Instrumentality **->** Loneliness **->** Wellbeing | 0.061 | -0.046, 0.195 | - | 0.026 |
|  | Indirect effect 3: Instrumentality **->** Connectedness **->** Loneliness **->** Wellbeing | -0.030 | -0.080, -0.008 | - | -0.013 |
| Total effect of Instrumentality Literacy on QoL (VAS) | | 1.420 | -1.692, 4.484 | .371 | 0.088 |
|  | Direct effect: Instrumentality **->** QoL (VAS) | 1.653 | -1.391, 4.658 | .290 | 0.103 |
|  | Indirect effect 1: Instrumentality **->** Connectedness **->** QoL (VAS) | -0.306 | -1.073, 0.068 | - | -0.019 |
|  | Indirect effect 2: Instrumentality **->** Loneliness **->** QoL (VAS) | 0.146 | -0.080, 0.692 | - | 0.009 |
|  | Indirect effect 3: Instrumentality **->** Connectedness **->** Loneliness **->** QoL (VAS) | -0.073 | -0.278, 0.001 | - | -0.005 |
| Total effect of Instrumentality Literacy on QoL (Utility) | | -0.002 | -0.046, 0.041 | .927 | -0.009 |
|  | Direct effect: Instrumentality **->** QoL (Utility) | 0.000 | -0.040, 0.038 | .997 | 0.000 |
|  | Indirect effect 1: Instrumentality **->** Connectedness **->** QoL (Utility) | -0.005 | -0.015, 0.000 | - | -0.023 |
|  | Indirect effect 2: Instrumentality **->** Loneliness **->** QoL (Utility) | 0.006 | -0.005, 0.019 | - | 0.027 |
|  | Indirect effect 3: Instrumentality **->** Connectedness **->** Loneliness **->** QoL (Utility) | -0.003 | -0.008, -0.001 | - | -0.013 |
| Total effect of Reassurance Literacy on Wellbeing | | -0.242 | -0.700, 0.194 | .292 | -0.076 |
|  | Direct effect: Reassurance **->** Wellbeing | -0.394 | -0.827, 0.024 | .069 | -0.124 |
|  | Indirect effect 1: Reassurance **->**Connectedness **->**Wellbeing | 0.091 | -0.029, 0.249 | - | 0.029 |
|  | Indirect effect 2: Reassurance **->**Loneliness **->**Wellbeing | 0.043 | -0.068, 0.165 | - | 0.014 |
|  | Indirect effect 3: Reassurance **->**Connectedness **->**Loneliness **->**Wellbeing | 0.018 | -0.004, 0.062 | - | 0.006 |
| Total effect of Reassurance Literacy on QoL (VAS) | | 0.702 | -2.551, 4.050 | .677 | 0.033 |
|  | Direct effect: Reassurance **->**QoL (VAS) | 0.368 | -2.887, 3.667 | .827 | 0.017 |
|  | Indirect effect 1: Reassurance **->**Connectedness **->**QoL (VAS) | 0.186 | -0.048, 0.894 | - | 0.009 |
|  | Indirect effect 2: Reassurance **->**Loneliness **->**QoL (VAS) | 0.104 | -0.106, 0.551 | - | 0.005 |
|  | Indirect effect 3: Reassurance **->**Connectedness **->**Loneliness **->**QoL (VAS) | 0.044 | -0.008, 0.221 | - | 0.002 |
| Total effect of Reassurance Literacy on QoL (Utility) | | -0.010 | -0.049, 0.029 | .631 | -0.033 |
|  | Direct effect: Reassurance **->**QoL (Utility) | -0.019 | -0.058, 0.020 | .349 | -0.064 |
|  | Indirect effect 1: Reassurance **->**Connectedness **->**QoL (Utility) | 0.003 | 0.000, 0.012 | - | 0.011 |
|  | Indirect effect 2: Reassurance **->**Loneliness **->**QoL (Utility) | 0.004 | -0.006, 0.016 | - | 0.014 |
|  | Indirect effect 3: Reassurance **->**Connectedness **->**Loneliness **->**QoL (Utility) | 0.002 | 0.000, 0.006 | - | 0.006 |
| Total effect of Pastime Literacy on Wellbeing | | -0.038 | -0.557, 0.488 | .887 | -0.015 |
|  | Direct effect: Pastime **->**Wellbeing | -0.085 | -0.513, 0.337 | .695 | -0.033 |
|  | Indirect effect 1: Pastime **->**Connectedness **->**Wellbeing | 0.116 | -0.029, 0.297 | - | 0.045 |
|  | Indirect effect 2: Pastime **->**Loneliness **->**Wellbeing | -0.092 | -0.286, 0.045 | - | -0.036 |
|  | Indirect effect 3: Pastime **->**Connectedness **->**Loneliness **->**Wellbeing | 0.024 | -0.003, 0.076 | - | 0.009 |
| Total effect of Pastime Literacy on QoL (VAS) | | -1.444 | -5.102, 2.203 | .447 | -0.083 |
|  | Direct effect: Pastime **->** QoL (VAS) | -1.518 | -5.108, 2.103 | .411 | -0.087 |
|  | Indirect effect 1: Pastime **->**Connectedness **->** QoL (VAS) | 0.239 | -0.054, 1.144 | - | 0.014 |
|  | Indirect effect 2: Pastime **->**Loneliness **->** QoL (VAS) | -0.222 | -1.091, 0.082 | - | -0.013 |
|  | Indirect effect 3: Pastime **->**Connectedness **->**Loneliness **->** QoL (VAS) | 0.057 | -0.009, 0.272 | - | 0.003 |
| Total effect of Pastime Literacy on QoL (Utility) | | -0.016 | -0.065, 0.030 | .505 | -0.068 |
|  | Direct effect: Pastime **->**QoL (Utility) | -0.013 | -0.057, 0.029 | .541 | -0.057 |
|  | Indirect effect 1: Pastime **->**Connectedness **->** QoL (Utility) | 0.004 | -0.001, 0.015 | - | 0.017 |
|  | Indirect effect 2: Pastime **->**Loneliness **->** QoL (Utility) | -0.009 | -0.027, 0.005 | - | -0.038 |
|  | Indirect effect 3: Pastime **->**Connectedness **->**Loneliness **->** QoL (Utility) | 0.002 | 0.000, 0.008 | - | 0.010 |
| ^a^ Wellbeing was measured using the Personal Wellbeing Score (PWS); QoL (Quality of Life; VAS) was measured using EQ VAS; QoL (Quality of Life; Utility) was measured using EQ-5D-3L which was converted to the Utility Index; Connectedness was measured using Lubben Social Network Scale (LSNS6); Loneliness was measured using UCLA3.  ^b^ Statistically significant (unstandardized) parameter estimate ($\hat{\beta}$) were underlined. Statistical significance was determined either using P-value (for total and direct effects) or 95% CI (for indirect effects). $\hat{\beta}_{std}$ represents the fully standardized parameter estimate. | | | | | |
