# Supplementary material for: Smartphone ownership, digital literacy, and the mediating role of social connectedness and loneliness in improving the wellbeing of community-dwelling older adults of low socio-economic status in Singapore
Source: PLoS One. 2023 Aug 30;18(8):e0290557. doi: 10.1371/journal.pone.0290557 (PMC10468072; doi:10.1371/journal.pone.0290557)
Supplement: Attachment — Submitted filename: PONE-D-23-04295_reviewer.pdf [file pone.0290557.s002.pdf]

PLOS ONE

Smartphone Ownership, Digital Literacy, and the Mediating Role of Connectedness and Loneliness in Improving the Wellbeing of Community-Dwelling Older Adults of Low Socio-Economic Status in Singapore

--Manuscript Draft--

**Manuscript Number:**	PONE-D-23-04295
**Article Type:**	Research Article
**Full Title:**	Smartphone Ownership, Digital Literacy, and the Mediating Role of Connectedness and Loneliness in Improving the Wellbeing of Community-Dwelling Older Adults of Low Socio-Economic Status in Singapore
**Short Title:**	Smartphones, Digital Literacy and the Wellbeing of Older Adults of Low Socioeconomic Status in Singapore
**Corresponding Author:**	Amrish Soundararajan<br>Trigen Ltd<br>Singapore, SINGAPORE
**Keywords:**	digital literacy; smartphones; social networks; social connectedness; social isolation; loneliness; wellbeing; quality of life; serial mediation analysis
**Abstract:**	During the COVID-19 pandemic, safe-distancing measures resulted in many community dwelling older adults being socially isolated and lonely. While digital technology may have mitigated this, older adults of low socioeconomic status (SES) who are not digitally literate remain vulnerable to the adverse effects of social isolation and loneliness. 302 community dwelling older adults of low SES were recruited to participate in a home-based digital literacy program to examine the relationship between digital literacy and social connectedness, loneliness, wellbeing, and quality of life (QoL). This study identified demographic factors associated with smartphone ownership, digital literacy, and willingness to enroll in a digital literacy education program. The 59.9% of participants who owned an internet-enabled smartphone tended to be younger and better educated. Older adults who were better educated, of Chinese descent (the ethnic majority in Singapore), and who had lower digital literacy despite owning smartphones, were most likely to enroll in the digital literacy education program. Social-use digital literacy had a positive indirect effect on measures of both wellbeing and QoL (mediated by social connectedness and perceived loneliness), while instrumental-use digital literacy had a negative indirect association with both measures. Further study is needed to evaluate whether interventions to improve digital literacy (especially for social-use) may help to reduce social isolation and loneliness, and ultimately improve wellbeing and QoL.
**Order of Authors:**	Amrish Soundararajan
	Jie Xin Lim
	Nerice Heng Wen Ngiam
	Angeline Jie Yin Tey
	Aaron Kai Wen Tang
	Haikel Lim
	Ka Shing Yow
	Ling Jie Cheng
	Jess Ho
	Nigel Qun Xuan Teo
	Wan Qi Yee
	Sungwon Yoon
	Lian Leng Low

Powered by Editorial Manager® and ProduXion Manager® from Aries Systems Corporation

	Kennedy Yao Yi Ng
**Additional Information:**	
**Question**	**Response**
**Financial Disclosure**<br><br>Enter a financial disclosure statement that describes the sources of funding for the work included in this submission. Review the submission guidelines for detailed requirements. View published research articles from *PLOS ONE* for specific examples.<br><br>This statement is required for submission and **will appear in the published article** if the submission is accepted. Please make sure it is accurate.<br><br>**Unfunded studies**<br>Enter: *The author(s) received no specific funding for this work.*<br><br>**Funded studies**<br>Enter a statement with the following details:<br>• Initials of the authors who received each award<br>• Grant numbers awarded to each author<br>• The full name of each funder<br>• URL of each funder website<br>• Did the sponsors or funders play any role in the study design, data collection and analysis, decision to publish, or preparation of the manuscript?<br>• **NO** - Include this sentence at the end of your statement: *The funders had no role in study design, data collection and analysis, decision to publish, or preparation of the manuscript.*<br>• **YES** - Specify the role(s) played.<br><br>* typeset	This research is supported by the Singapore Ministry of Health's National Medical Research Council under the Fellowship Programme by SingHealth Regional Health System, Population-based, Unified, Learning System for Enhanced and Sustainable (PULSES) Health Centre Grant (NMRC/CG/C027/2017_SHS), the Healthy, Empowered and Active Living (HEAL) fund and the Infocomm Media Development Authority Digital for Life Fund. The funders had no role in study design, data collection and analysis, decision to publish, or preparation of the manuscript.
**Competing Interests**<br><br>Use the instructions below to enter a competing interest statement for this submission. On behalf of all authors, disclose any competing interests that could be perceived to bias this work—acknowledging all financial support and any other relevant financial or non-financial competing interests.	The authors have declared that no competing interests exist.

This statement is required for submission and **will appear in the published article** if the submission is accepted. Please make sure it is accurate and that any funding sources listed in your Funding Information later in the submission form are also declared in your Financial Disclosure statement.<br><br>View published research articles from *PLOS ONE* for specific examples.<br><br>**NO authors have competing interests**<br><br>Enter: *The authors have declared that no competing interests exist.*<br><br>**Authors with competing interests**<br><br>Enter competing interest details beginning with this statement:<br><br>*I have read the journal's policy and the authors of this manuscript have the following competing interests: [insert competing interests here]*<br><br>* typeset	
**Ethics Statement**<br><br>Enter an ethics statement for this submission. This statement is required if the study involved:<br><br>• Human participants<br>• Human specimens or tissue<br>• Vertebrate animals or cephalopods<br>• Vertebrate embryos or tissues<br>• Field research<br><br>Write "N/A" if the submission does not require an ethics statement.<br><br>General guidance is provided below. Consult the submission guidelines for detailed instructions. **Make sure that all**	This study was approved by SingHealth Centralised Institutional Review Board - Reference number: 2020/2722.<br>Written consent was obtained for participants who were keen to enroll in the digital literacy education program, and verbal consent was obtained for those who declined enrollment in the digital literacy education program.

Powered by Editorial Manager® and ProduXion Manager® from Aries Systems Corporation

information entered here is included in the Methods section of the manuscript.<br><br>**Format for specific study types**<br><br>**Human Subject Research (involving human participants and/or tissue)**<br>• Give the name of the institutional review board or ethics committee that approved the study<br>• Include the approval number and/or a statement indicating approval of this research<br>• Indicate the form of consent obtained (written/oral) or the reason that consent was not obtained (e.g. the data were analyzed anonymously)<br><br>**Animal Research (involving vertebrate animals, embryos or tissues)**<br>• Provide the name of the Institutional Animal Care and Use Committee (IACUC) or other relevant ethics board that reviewed the study protocol, and indicate whether they approved this research or granted a formal waiver of ethical approval<br>• Include an approval number if one was obtained<br>• If the study involved *non-human primates*, add *additional details* about animal welfare and steps taken to ameliorate suffering<br>• If anesthesia, euthanasia, or any kind of animal sacrifice is part of the study, include briefly which substances and/or methods were applied<br><br>**Field Research**<br><br>Include the following details if this study involves the collection of plant, animal, or other materials from a natural setting:<br>• Field permit number<br>• Name of the institution or relevant body that granted permission	
**Data Availability**<br><br>Authors are required to make all data underlying the findings described fully available, without restriction, and from the time of publication. PLOS allows rare exceptions to address legal and ethical	Yes - all data are fully available without restriction

concerns. See the PLOS Data Policy and FAQ for detailed information.<br><br>A Data Availability Statement describing where the data can be found is required at submission. Your answers to this question constitute the Data Availability Statement and **will be published in the article**, if accepted.<br><br>**Important:** Stating 'data available on request from the author' is not sufficient. If your data are only available upon request, select 'No' for the first question and explain your exceptional situation in the text box.<br><br>Do the authors confirm that all data underlying the findings described in their manuscript are fully available without restriction?	
**Describe where the data may be found in full sentences. If you are copying our sample text, replace any instances of XXX with the appropriate details.**<br><br>• If the data are **held or will be held in a public repository**, include URLs, accession numbers or DOIs. If this information will only be available after acceptance, indicate this by ticking the box below. For example: *All XXX files are available from the XXX database (accession number(s) XXX, XXX.*).<br>• If the data are all contained **within the manuscript and/or Supporting Information files**, enter the following: *All relevant data are within the manuscript and its Supporting Information files.*<br>• If neither of these applies but you are able to provide **details of access elsewhere**, with or without limitations, please do so. For example:<br><br>*Data cannot be shared publicly because of [XXX]. Data are available from the XXX Institutional Data Access / Ethics Committee (contact via XXX) for researchers who meet the criteria for access to confidential data.*	DOI: 10.17605/OSF.IO/YVSHJ

The data underlying the results presented in the study are available from (include the name of the third party and contact information or URL).<br>• This text is appropriate if the data are owned by a third party and authors do not have permission to share the data.<br><br>* typeset	
Additional data availability information:	Tick here if the URLs/accession numbers/DOIs will be available only after acceptance of the manuscript for publication so that we can ensure their inclusion before publication.

Powered by Editorial Manager® and ProduXion Manager® from Aries Systems Corporation

Manuscript
Click here to access/download;Manuscript;PlosOne Manuscript.docx

Smartphone Ownership, Digital Literacy, and the Mediating Role of Connectedness and Loneliness in Improving the Wellbeing of Community-Dwelling Older Adults of Low Socio-Economic Status in Singapore

Amrish Soundararajan[1,2¶], Jie Xin Lim[1,3¶], Nerice Heng Wen Ngiam[1,4,5¶], Angeline Jie-Yin Tey[1,5,6], Aaron Kai Wen Tang[1,5,7], Haikel A. Lim[1,8,12], Ka Shing Yow[1,9], Ling Jie Cheng[1,10], Jess Ho[11], Qun Xuan Nigel Teo[5], Wan Qi Yee[5], Sungwon Yoon[12,13], Lian Leng Low[5,13,14], Kennedy Yao Yi Ng [1,5,15*]

[1] TriGen, Singapore
[2] Department of Family Medicine, National University Health System (NUHS), Singapore
[3] Wee Kim Kee School of Communication and Information, Nanyang Technological University, Singapore
[4] Department of Internal Medicine, Singapore General Hospital, Singapore
[5] Population Health and Integrated Care Office, Singapore General Hospital, Singapore
[6] Department of Internal Medicine, Tan Tock Seng Hospital, Singapore
[7] Department of Psychiatry, Singapore General Hospital, Singapore
[8] Department of Psychiatry, National Health Group, Singapore
[9] Department of Internal Medicine, National University Health System, Singapore
[10] Health Systems and Behavioral Sciences Domain, Saw See Hock School of Public Health, National University of Singapore, Singapore

[11]NTUC Health Co-operative Ltd, Singapore

[12] Health Services and Systems Research, Duke-NUS Medical School, Singapore

[13] Centre for Population Health Research and Implementation, SingHealth Regional Health System

[14] SingHealth Duke-NUS Family Medicine Academic Clinical Program, Duke-NUS Medical School, Singapore

[15] Division of Medical Oncology, National Cancer Centre Singapore, Singapore

*** Corresponding Author:**

Email: kennedy.ng.y.y@nccs.com.sg

¶These authors contributed equally to this work

Abstract

During the COVID-19 pandemic, safe-distancing measures resulted in many community dwelling older adults being socially isolated and lonely. While digital technology may have mitigated this, older adults of low socioeconomic status (SES) who are not digitally literate remain vulnerable to the adverse effects of social isolation and loneliness. 302 community dwelling older adults of low SES were recruited to participate in a home-based digital literacy program to examine the relationship between digital literacy and social connectedness, loneliness, wellbeing, and quality of life (QoL). This study identified demographic factors associated with smartphone ownership, digital literacy, and willingness to enroll in a digital literacy education program. The 59.9% of participants who owned an internet-enabled smartphone tended to be younger and better educated. Older adults who were better educated, of Chinese descent (the ethnic majority in Singapore), and who had lower digital literacy despite owning smartphones, were most likely to enroll in the digital literacy education program. Social-use digital literacy had a positive indirect effect on measures of both wellbeing and QoL (mediated by social connectedness and perceived loneliness), while instrumental-use digital literacy had a negative indirect association with both measures. Further study is needed to evaluate whether interventions to improve digital literacy (especially for social-use) may help to reduce social isolation and loneliness, and ultimately improve wellbeing and QoL.

Introduction

Social connectedness (objective presence or absence of social contacts) and loneliness (subjective feeling resulting from social isolation) have long been recognized as key social determinants of health (1) such as depression (2-4), low quality of life, lack of wellbeing (5), morbidity (6), and mortality (7). The two concepts, while distinct, are interrelated (8). For example, loneliness has been found to mediate the effect of social connectedness on wellbeing (5).

Safe-distancing measures were essential to the COVID-19 pandemic control policies globally (9, 10) to lower the rate of transmission. However, these measures were also associated with adverse consequences such as increasing social isolation and loneliness (11).

The Role of Information and Communications Technology

To overcome the physical barriers associated with safe-distancing measures and the associated negative consequences, the use of information and communications technology (ICT) have been employed. Singapore has sped up the implementation of its Digital Transformation Master Plan during the pandemic (12). The use of ICT, through devices like smartphones, has potential to help older adults maintain social connectedness and reduce loneliness (13). A pre-pandemic study suggested that ICT use among older adults is associated with fewer depressive symptoms, higher self-rated health, and subjective wellbeing, with these relationships being mediated by reduced loneliness (14). An improved digital literacy would enable older adults to extend their social connections and receive support from these connections (15); ICT could therefore mitigate the negative effects of emotional and social loneliness caused by the safe-distancing measures implemented during the COVID-19 pandemic (16).

Addressing the Digital Divide

While there are significant benefits to the use of ICT amongst older adults during a pandemic; there undoubtedly remains a significant digital divide with the older adult less likely to access ICT (17). This issue is multifactorial, ranging from health-related reason (18) to reasons such as negative beliefs or no perceived benefits of ICT, low perceived ability and low digital literacy (17). Low-income and lower education levels are also identified as key deterrents of ICT usage. Furthermore, the presence of social support to support older adults in their use of ICT seems to be important (19). As such, older adults of lower socio-economic status (SES) and those with minimal social support are likely to be digitally illiterate and vulnerable to the adverse outcomes of safe-distancing measures during the COVID-19 pandemic, and this may be mediated by greater loneliness and social isolation (20).

The Present Study

In response to this digital divide, the Infocomm Media Development Authority Singapore (IMDA) launched the Seniors Go Digital Program, aiming to increase digital literacy among older adults living in Singapore (21). The program also addresses access issues by providing subsidized smartphones and data subscriptions to seniors of low SES. TriGen, a charity, collaborated with IMDA and senior activity centers to bring a home-based digital literacy program (DLP) to these individuals. The study primarily aimed to understand the factors that affect the propensity of the DLP participation.

Secondly, this study aimed to test the hypothesized relations between digital literacy, connectedness, loneliness, and wellbeing and quality of life. As referenced above, ICT use helps older adults maintain social connectedness, and reduces loneliness. Given that loneliness and social connectedness are established social determinants of health and well-being, and that the higher connectedness is expected to lower the sense of loneliness, it was hypothesized that the effect of digital literacy on the wellbeing and quality of life of older

adults would be serially mediated by social connectivity and loneliness. On a more granular level, it was hypothesized that the mediation effect may be particularly evident for domains of digital literacy pertaining specifically to social interaction.

Materials and Method

The study was reported following the Strengthening the Reporting of Observational Studies in Epidemiology (STROBE) guidelines for cross-sectional studies.

Participants and Procedure

A cross-sectional study was carried out between August 2020 and June 2021. The inclusion criteria for the study were as follows: residents in the South-West region of Singapore; aged >55 years; belonging to lower SES (residing in public rental housing or receiving welfare payments from the Public Assistance Scheme); and who consented to partake in this study (regardless of enrolment in the home-based digital literacy program; DLP). Our study reached out to these individuals by working with eldercare and welfare service providers in the community.

Recruitment of participants involved phone calls and door-to-door outreach. Written consent was obtained for older adults who were keen to participate in the DLP and verbal consent was obtained for those who declined enrollment in the DLP but agreed to partake in this research study.

Data was collected from participants either in person or via telephone through standardized questionnaires administered in the participants' preferred language. Standardized training was provided for surveyors prior to household visits for recruitment and survey administration. If there was no response at the first instance, the visit or phone call was conducted on at least three separate occasions, with at least one scheduled on a weekend, to maximize participation.

Instruments

There is no universally accepted definition of digital literacy (22). Hence, digital literacy was defined based on the Singapore Digital Readiness Blueprint, which refers to digital literacy as the ability to confidently use digital technologies (23). To measure digital literacy, a questionnaire was specifically constructed to quantify the extent to which the participants use 13 specific functions of an internet-enabled phone (smartphone) based on four aspects of digital literacy: sociability, instrumentality, reassurance, and pastime based on existing literature (24, 25). Refer to Table S1 in S1 Appendix for the questionnaire. Sociability refers to the use of functions or applications for relationship maintenance; Instrumentality refers to the fulfilment of practical activities of daily life; Reassurance refers to the ability to obtain help in an emergency; and Pastime refers to using the smartphone as an entertainment device (25).

Social connectedness was measured using the Lubben Social Network Scale (LSNS-6), where a total summed score of <12 suggests that participants are at risk of social isolation (26). Perceived loneliness was assessed using the UCLA 3-item loneliness scale (UCLA-3; 27); wellbeing was assessed using the Personal Wellbeing Score (PWS) (28) where a higher score indicates better wellbeing. Quality of life was assessed using the EQ-5D-3L and EQ VAS (29). Value set derived from a local population study was used to calculate a EQ-5D-3L utility index (30).

Data Analysis

Monte Carlo simulations suggested a minimum sample size of 300 was required to achieve a maximum Type I error rate of 0.05 and Type II error rate of 0.20 for most parameters under the assumption of small effect sizes (standardized $\hat{\beta} = 0.20$) for the serial mediation model.

Principal Component Analysis (PCA) was used to evaluate the composition of the digital literacy index for each of the four aspects of digital literacy. PCA is a common methodology to create composite indices such as the social-economic status (31). A square matrix of the bivariate Phi coefficient (i.e., a special case of Pearson's correlation coefficient for dichotomous data) of the 13 smartphone features utilization (1 = yes, 0 = no) was computed for the analyses using a subset of samples with complete observations (n = 266). PCA was conducted using the pre-determined features utilization for each of the four literacy indices. The four composite indices scores were summed using the dichotomized responses of the respective phone functions. An overall digital literacy index score was computed by summing the four composite indices scores. Scores for connectedness, loneliness, and wellbeing were computed by summing the item ratings. The EQ-5D-3L utility index was computed using value set derived from the local population (30) as implemented in the eq5d R package (32).

Descriptive statistics and binary logistic regression were conducted using Python 3.7.11 on Google Colaboratory. Univariable analysis was performed to find the associations between socio-demographic variables, and digital literacy, smartphone ownership, as well as participants' propensity to enroll in a DLP respectively. Multivariable binary logistic regression models were built on the univariable analyses, to predict the adjusted effects of selected socio-demographic variables. Per-analysis listwise deletion was utilized for instances of missing data.

The path relationship between digital literacy (sociability, instrumentality, reassurance, and pastime), connectedness, loneliness, wellbeing, and quality of life was modelled using a serial mediation model in Mplus v8.4 (Figure 1). Full Information Maximum Likelihood was used to accommodate the missing data in the endogenous variables under the missing at random mechanism. A series of simple linear regression

models with connectedness, loneliness, wellbeing, and quality of life as the univariate outcomes regressed on each demographic variable were conducted to identify control variables to be included in the mediation model. Gender was found to be associated with Connectedness and age was found to the associated with quality of life (EQ-5D-3L utility index). These two variables were therefore included in the model to partial out their effect on the respective variable. Age and smartphone ownership were also independently found to be statistically significant predictors of the presence of missing data on the four literacy indices (0 = missing observation, 1 = observation available) using a series of logistic regressions. Hence these two variables were included in the model as auxiliary variables (predictors) of the literacy indices to account for the presence of missing data.

Indirect effects representing the mediation processes were tested using bias-corrected and accelerated (BCa) bootstrap 95% confidence interval with 5000 replications. Model-data fit was evaluated using Hu and Bentler's (33) strategy (i.e., $SRMR \leq 0.08$, $CFI \geq 0.95$, and $RMSEA \leq 0.05$). The nominal α level of 0.05 was used to evaluate all null hypothesis significance testing.

Results

Demographics

A total of 366 older adults were referred. 64 rejected participation in the research or could not be contacted, leaving a total of 302 older adults (82.5%) in the study. The median age is 73, 55% were female, with a representative ethnic mix similar to the general local population (34). 75.5% had at most 6 years of formal education; 83.4% stayed in public rental housing (applicants' gross household income equivalent to <USD1100), and 56.6% stayed in one-room apartments (the smallest units in Singapore's public housing developments) suggestive of lower SES. 80.5% of participants already had a mobile phone at the time of the

survey (59.9% had smartphones). Almost half (49%) of the participants agreed to enroll in the home-based digital literacy program (DLP; Table 1).

Table 1. Demographic characteristics of the participants.

**Variables**		**Values**
**Age (years); median (IQR)**		73 (13)
**Gender, n (%)**		
	Female	167 (55.3)
	Male	135 (44.7)
**Race, n (%)**		
	Chinese	234 (77.5)
	Malay	40 (13.2)
	Indian	26 (8.6)
	Others	2 (0.7)
**Highest Level of Education[a], n (%)**		
	No formal education	102 (33.8)
	Primary education	126 (41.7)
	Secondary education	66 (21.9)
	Tertiary education	5 (1.7)
**Housing Type[b], n (%)**		
	Rented	252 (83.4)
	Owned (99-year leasehold)	45 (14.9)
**Apartment Size[c], n (%)**		
	One room	171 (56.6)

	Two room	104 (34.4)
	Three room	21 (7.0)
	Four room	5 (1.7)
**Mobile Phone Ownership, n (%)**		
	Yes	243 (80.5)
	No	59 (19.5)
**Smartphone Ownership, n (%)**		
	Yes	181 (59.9)
	No	121 (40.1)
**Joined Project Wire-up, n (%)**		
	Yes	148 (49.0)
	No	154 (51.0)

[a] n = 3 (1.0%) were unsure of their education level.

[b] n = 5 (1.7%) were unsure of their housing type.

[c] n = 1 (0.3%) did not indicate the category.

PCA of Digital Literacy Survey Items

PCA was used to evaluate the construction of the literacy indices (sociability, instrumentality, reassurance, and pastime literacy index). Parallel analyses suggested the extraction of one component in each PCA. Subsequent PCA showed that at least 65% of the total variance can be explained by the one-component solution for each of the indices and the component weights were all above 0.60 (see Table 2 for the combined PCA results). The results provided the evidence for an equal weighting of the item responses to compute each of the composite index score.

Table 2. Results of Principal Component Analyses on the Digital Literacy Survey Items[a]

Items	Sociability	Instrumentality	Reassurance	Pastime
Text Message	0.85			
Video Call	0.83			
Voice Call	0.75			
Government Apps		0.90		
Online Banking		0.87		
Online Purchase		0.84		
Health Apps		0.81		
Read News		0.61		
Call Ambulance			0.97	
Call Police			0.97	
Listen to Music				0.90
Watch Video				0.89
Play Games				0.80
Variance Explained	66%	66%	94%	74%

[a] Each column (component) represents results from individual principal component analysis (PCA). Values in the cells represent the PCA component weights.

Digital Literacy, Loneliness, Connectedness, Wellbeing and Quality of Life

The overall digital literacy amongst the participants was low, with a median score of 3 (observed minimum of 0 and maximum of 13). Participants in general reported low loneliness (median score of 3 on an observed range of 3 to 9) and wellbeing (median score of 8 on an

observed range of 0 to 12). 168 (55.6%) of the participants were at risk of social isolation (LSNS6 < 12; 22). The median EQ-5D-3L utility index was 0.854 and the median EQ VAS was 70. Descriptive statistics of the key measurements can be found in Table S2 in S1 Appendix. Participants who owned mobile phones reported better connectedness (measured using LSNS6) than those without mobile phones (median 11 vs. 8, $p = .002$), as well as better quality of life (median EQ-5D-3L utility index 0.854 vs 0.832, $p = .02$).

Factors Associated with Digital Literacy, Phone Ownership and Propensity to Enroll in a Digital Literacy Program

Comparing participants with an overall digital literacy index ≤ 3 (i.e., the median) with those with an overall digital literacy index > 3, logistic regression modeling (Table S3 in S1 Appendix) showed that female (compared to male) was independently associated with poorer digital literacy (adjusted odds-ratio [aOR] = 0.49, 95% CI = [0.28. 0.85], $p = .011$) and was of an older age (aOR = 0.99, 95% CI = [0.98, 1.00], $p = .048$). Higher education levels (using absence of formal education as the reference group) were independently associated with better digital literacy (Primary education aOR = 2.47, 95% CI = [1.36, 4.49], $p = .003$; Secondary and Tertiary education aOR = 6.17, 95% CI = [2.98, 12.74], $p < .001$).

Higher education levels also independently increased the odds of smartphone ownership (aOR for Primary education = 2.37, 95% CI = [1.42, 3.98], $p = .001$; aOR for Secondary and Tertiary education = 5.41, 95% CI = [2.86, 10.62], $p < .001$). Other socio-demographic factors (gender, race, housing type, apartment size) did not have any statistically significant association, with age being of borderline significance (Table S4 in S1 Appendix).

There was no statistically significant difference in the median ages between those who wished to enroll in the DLP and those who did not. Adjusted using logistic regression, higher education levels (compared to no formal education; aOR for Primary education = 1.47, 95%

CI = [0.83, 2.61], p = .190; aOR for Secondary and Tertiary education = 2.63, 95% CI = [1.23, 5.62], p = .012) and prior smartphone ownership (aOR = 4.90, 95% CI = [2.37, 10.13], $p < .001$) independently increased the odds of a participant being willing to enroll in a DLP. Meanwhile lower digital literacy index (aOR = 0.86, 95% CI = [0.78, 0.94], $p = .002$) and being Chinese (compared to non-Chinese; aOR = 0.45, 95% CI= [0.29, 0.70], $p < .001$) decreased the odds of being willing to enroll (Table 3).

Table 3. Factors associated with willingness to join a home-based mobile digital literacy education program.

	Willingness to enroll in Digital Literacy Improvement Program		Univariable Tests	Multivariable Logistic Regression[a]	
**Variables**	**No (n = 154)**	**Yes (n = 148)**	**P-value[b]**	**Odds Ratio [95% CI]**	**P-value**
**Age (year), Median (IQR)**	73 (13)	73 (14)	.19**	-	-
**Gender, n (%)**					
Female	80 (47.9)	87 (52.1)	.28*	-	-
Male	74 (54.8)	61 (45.2)		-	-
**Race, n (%)**					
Chinese	128 (54.7)	106 (45.3)	.02*	0.45 [0.29, 0.70]	< .001
Non- Chinese	26 (38.2)	42 (61.8)		-[c]	-
**Education Level, n (%)**					
No formal education	62 (60.8)	40 (39.2)	.03*	-[c]	-
Primary education	63 (50.0)	63 (50.0)		1.47 [0.83, 2.61]	.19

Secondary and Tertiary education	29 (40.1)	42 (59.2)				2.63 [1.23, 5.62]	.01
**Housing Type, n (%)**							
Rented	138 (54.8)	114 (45.2)		.01*		-[c]	-
Owned	15 (33.3)	30 (66.7)				2.14 [0.99, 4.63]	.055
**Apartment Size, n (%)**							
One room	92 (53.8)	79 (46.2)		.31*		-	-
Two room	51 (49.0)	53 (51.0)				-	-
Larger than Two room	10 (38.5)	16 (61.5)				-	-
**Prior smartphone, n (%)**							
No	78 (65.5)	41 (34.5)		< .001*		-[c]	-
Yes	75 (41.4)	106 (58.6)				4.90[2.37-10.13]	< .001
**Digital Literacy Index, Median (IQR)**	3 (7)	4 (6)		.03**		0.86 [0.78-0.94]	.002

[a] Only variables with statistically significant association found in the univariable tests were included in the logistic regression model. After listwise exclusion of those with missing data, 259 participants were included in the final regression model.

[b] *Chi-squared Test. **Mann-Whitney U-Test.

[c] Reference group of the variable.

Association between Digital Literacy, Connectedness, Loneliness, Wellbeing, and Quality of Life

Serial mediation modeling (SMM) was performed to test the hypothesized association between the digital literary indices, connectedness ($\alpha = 0.80$), loneliness ($\alpha = 0.87$), wellbeing ($\alpha = 0.86$), and quality of life. Internal consistency (Cronbach's α) for the digital literacy scale was not estimated because it was a constructed formative measure as opposed to a reflective measure. We were unable to estimate the test-retest reliability from this cross-sectional data. For the same reason, estimation of the test-retest reliability of the EQ-5D-3L utility index and EQ VAS (quality of life) was not possible.

SMM partials the total effect of a predictor on an outcome into two components: (1) direct effect and (2) indirect effects. For an instance, in this specific model, the total association between social digital literacy on wellbeing can be partitioned into (1) a direct effect of social digital literacy on wellbeing, (2a) a specific indirect effect via connectedness, (2b) a specific indirect effect via loneliness, and (2c) a specific indirect effect via connectedness and loneliness. See Fig 1 and Table 4 for a depiction and the results of the serial mediation model.

Fig 1. A simplified representation of the serial mediation model and the unstandardized regression weights.

$b \quad a1(\cdot)$ represents the effect of social literacy on the connectedness (m1) or loneliness (m2), while $b \quad a2(\cdot)$, $b \quad a3(\cdot)$, $b \quad a4(\cdot)$and represent the effect of instrumental literacy, reassurance literacy, and pastime literacy respectively. Similarly, y1, y2, and y3 in $b \quad b1(\cdot)$ and $b \quad b2(\cdot)$ represents each of the three outcomes: wellbeing, quality of life (EQ-VAS), and

quality of life (EQ-5D-3L Utility Index). $\chi 2(22) = 36.333, p = .03$, RMSEA = .05, CFI = .99, SRMR = .04.

Table 4. Results of Serial Mediation Model[a, b]

Total, Direct, and Indirect Effects		$\hat{\boldsymbol{\beta}}$	95% CI	P-value	$\hat{\boldsymbol{\beta}}_{std}$
Total effect of Social Literacy on Wellbeing		<u>0.648</u>	0.111, 1.147	.016	0.238
	Direct effect: Social -> Wellbeing	0.367	-0.144, 0.816	.133	0.135
	Indirect effect 1: Social -> Connectedness -> Wellbeing	<u>0.181</u>	0.032, 0.393	-	0.066
	Indirect effect 2: Social -> Loneliness -> Wellbeing	0.063	-0.092, 0.239	-	0.023
	Indirect effect 3: Social -> Connectedness -> Loneliness -> Wellbeing	<u>0.037</u>	0.007, 0.101	-	0.013
Total effect of Social Literacy on QoL (VAS)		2.369	-1.030, 5.848	.178	0.128
	Direct effect: Social -> QoL (VAS)	1.756	-1.620, 5.198	.313	0.095
	Indirect effect 1: Social -> Connectedness -> QoL (VAS)	0.372	-0.071, 1.317	-	0.020
	Indirect effect 2: Social -> Loneliness -> QoL (VAS)	0.152	-0.160, 0.888	-	0.008
	Indirect effect 3: Social -> Connectedness -> Loneliness -> QoL (VAS)	0.088	-0.001, 0.339	-	0.005
Total effect of Social Literacy on QoL (Utility)		0.043	-0.013, 0.095	.114	0.174
	Direct effect: Social -> QoL (Utility)	0.027	-0.023, 0.075	.270	0.111
	Indirect effect 1: Social -> Connectedness -> QoL (Utility)	0.006	0.000, 0.018	-	0.025
	Indirect effect 2: Social -> Loneliness -> QoL (Utility)	0.006	-0.009, 0.023	-	0.024

	Indirect effect 3: Social -> Connectedness -> Loneliness -> QoL (Utility)	<u>0.004</u>	0.001, 0.010	-	0.014
Total effect of Instrumentality Literacy on Wellbeing		-0.113	-0.552, 0.335	.618	-0.048
	Direct effect: Instrumentality -> Wellbeing	0.005	-0.446, 0.417	.981	0.002
	Indirect effect 1: Instrumentality -> Connectedness -> Wellbeing	<u>-0.149</u>	-0.309, -0.034	-	-0.063
	Indirect effect 2: Instrumentality -> Loneliness -> Wellbeing	0.061	-0.046, 0.195	-	0.026
	Indirect effect 3: Instrumentality -> Connectedness -> Loneliness -> Wellbeing	<u>-0.030</u>	-0.080, -0.008	-	-0.013
Total effect of Instrumentality Literacy on QoL (VAS)		1.420	-1.692, 4.484	.371	0.088
	Direct effect: Instrumentality -> QoL (VAS)	1.653	-1.391, 4.658	.290	0.103
	Indirect effect 1: Instrumentality -> Connectedness -> QoL (VAS)	-0.306	-1.073, 0.068	-	-0.019
	Indirect effect 2: Instrumentality -> Loneliness -> QoL (VAS)	0.146	-0.080, 0.692	-	0.009
	Indirect effect 3: Instrumentality -> Connectedness -> Loneliness -> QoL (VAS)	-0.073	-0.278, 0.001	-	-0.005
Total effect of Instrumentality Literacy on QoL (Utility)		-0.002	-0.046, 0.041	.927	-0.009
	Direct effect: Instrumentality -> QoL (Utility)	0.000	-0.040, 0.038	.997	0.000
	Indirect effect 1: Instrumentality -> Connectedness -> QoL (Utility)	-0.005	-0.015, 0.000	-	-0.023
	Indirect effect 2: Instrumentality -> Loneliness -> QoL (Utility)	0.006	-0.005, 0.019	-	0.027

	Indirect effect 3: Instrumentality -> Connectedness -> Loneliness -> QoL (Utility)	-0.003	-0.008, -0.001	-	-0.013
Total effect of Reassurance Literacy on Wellbeing		-0.242	-0.700, 0.194	.292	-0.076
	Direct effect: Reassurance -> Wellbeing	-0.394	-0.827, 0.024	.069	-0.124
	Indirect effect 1: Reassurance ->Connectedness ->Wellbeing	0.091	-0.029, 0.249	-	0.029
	Indirect effect 2: Reassurance ->Loneliness ->Wellbeing	0.043	-0.068, 0.165	-	0.014
	Indirect effect 3: Reassurance ->Connectedness ->Loneliness ->Wellbeing	0.018	-0.004, 0.062	-	0.006
Total effect of Reassurance Literacy on QoL (VAS)		0.702	-2.551, 4.050	.677	0.033
	Direct effect: Reassurance ->QoL (VAS)	0.368	-2.887, 3.667	.827	0.017
	Indirect effect 1: Reassurance ->Connectedness ->QoL (VAS)	0.186	-0.048, 0.894	-	0.009
	Indirect effect 2: Reassurance ->Loneliness ->QoL (VAS)	0.104	-0.106, 0.551	-	0.005
	Indirect effect 3: Reassurance ->Connectedness ->Loneliness ->QoL (VAS)	0.044	-0.008, 0.221	-	0.002
Total effect of Reassurance Literacy on QoL (Utility)		-0.002	-0.049, 0.029	.927	-0.033
	Direct effect: Reassurance ->QoL (Utility)	-0.019	-0.058, 0.020	.349	-0.064
	Indirect effect 1: Reassurance ->Connectedness ->QoL (Utility)	-0.005	0.000, 0.012	-	0.011
	Indirect effect 2: Reassurance ->Loneliness ->QoL (Utility)	0.006	-0.006, 0.016	-	0.014

	Indirect effect 3: Reassurance ->Connectedness ->Loneliness ->QoL (Utility)	-0.003	0.000, 0.006	-	0.006
Total effect of Pastime Literacy on Wellbeing		-0.038	-0.557, 0.488	.887	-0.015
	Direct effect: Pastime ->Wellbeing	-0.085	-0.513, 0.337	.695	-0.033
	Indirect effect 1: Pastime ->Connectedness ->Wellbeing	0.116	-0.029, 0.297	-	0.045
	Indirect effect 2: Pastime ->Loneliness ->Wellbeing	-0.092	-0.286, 0.045	-	-0.036
	Indirect effect 3: Pastime ->Connectedness ->Loneliness ->Wellbeing	0.024	-0.003, 0.076	-	0.009
Total effect of Pastime Literacy on QoL (VAS)		-1.444	-5.102, 2.203	.447	-0.083
	Direct effect: Pastime -> QoL (VAS)	-1.518	-5.108, 2.103	.411	-0.087
	Indirect effect 1: Pastime ->Connectedness -> QoL (VAS)	0.239	-0.054, 1.144	-	0.014
	Indirect effect 2: Pastime ->Loneliness -> QoL (VAS)	-0.222	-1.091, 0.082	-	-0.013
	Indirect effect 3: Pastime ->Connectedness ->Loneliness -> QoL (VAS)	0.057	-0.009, 0.272	-	0.003
Total effect of Pastime Literacy on QoL (Utility)		-0.016	-0.065, 0.030	.505	-0.068
	Direct effect: Pastime ->QoL (Utility)	-0.013	-0.057, 0.029	.541	-0.057
	Indirect effect 1: Pastime ->Connectedness -> QoL (Utility)	0.004	-0.001, 0.015	-	0.017
	Indirect effect 2: Pastime ->Loneliness -> QoL (Utility)	-0.009	-0.027, 0.005	-	-0.038
	Indirect effect 3: Pastime ->Connectedness ->Loneliness -> QoL (Utility)	0.002	0.000, 0.008	-	0.010

[a] Wellbeing was measured using the Personal Wellbeing Score (PWS); QoL (Quality of Life; VAS) was measured using EQ VAS; QoL (Quality of Life; Utility) was measured using EQ-5D-3L which was converted to the Utility Index; Connectedness was measured using Lubben Social Network Scale (LSNS6); Loneliness was measured using UCLA3.

[b] Statistically significant (unstandardized) parameter estimate ($\hat{\beta}$) were underlined. Statistical significance was determined either using P-value (for total and direct effects) or 95% CI (for indirect effects). $\hat{\beta}_{std}$ represents the fully standardized parameter estimate.

A differential effect of the type of digital literacy on the outcomes was found. Social digital literacy had a statistically significant positive total effect on wellbeing ($\hat{\beta} = 0.65, p = .02$). Two associated specific indirect effects were found to be statistically significant. The effect of social digital literacy was found to be mediated by connectedness ($\hat{\beta} = 0.18, 95\%\ CI = [0.03, 0.39]$). The effect was also serially mediated via connectedness and loneliness ($\hat{\beta} = 0.04, 95\%\ CI = [0.01, 0.10]$). This set of results suggested an increase in social digital literacy is associated with an increase wellbeing as a result of the impact of social digital literacy on improving connectedness ($\hat{\beta} = 1.341$) and the impact of connectedness on decreasing loneliness ($\hat{\beta} = -0.051$) which in turn improved the reported sense of wellbeing ($|\hat{\beta}| = 0.538$).

A positive serial mediation of connectedness through loneliness on quality of life (EQ-5D-3L utility index) was also found for social digital literacy ($\hat{\beta} = 0.004, 95\%\ CI = [0.001, 0.010]$). This set of results suggested that social digital literacy improved connectedness which in turn decreased loneliness and a decreased loneliness would subsequently improve the quality of life ($|\hat{\beta}| = 0.052$).

In contrast, while instrumental digital literacy did not have a statistically significant total effect on wellbeing, it had a negative indirect effect on wellbeing via connectedness ($\hat{\beta} = -0.15, 95\%\ CI = [-0.31, -0.03]$). The effect was also serially mediated via connectedness through loneliness ($\hat{\beta} = -0.03, 95\%\ CI = [-0.08, -0.01]$). This set of result suggested that a higher instrumental digital literacy is associated with a lower connectedness ($\hat{\beta} = -1.104$), which in turn affected wellbeing ($\hat{\beta} = 0.135$). Simultaneously, the lowered connectedness also increased loneliness ($\hat{\beta} = -0.051$) which in turn decreased wellbeing ($\hat{\beta} = -0.538$).

A negative serial mediation of connectedness through loneliness on quality of life (EQ-5D-3L utility index) was also found for instrumental digital literacy ($\hat{\beta} = -0.003, 95\%\ CI = [-0.008\ , -0.001]$). This set of results suggested that instrumental digital literacy decreased connectedness, which in turn increased loneliness, and an increased loneliness would be detrimental to the quality of life ($\hat{\beta} = -0.052$).

In contrast to the impact of social and instrumental digital literacy, reassurance digital literacy and pastime digital literacy did not have any statistically significant association with the outcomes of interest, both directly or indirectly.

Discussion

Examining prevailing patterns of phone ownership and digital literacy, this study found that 59.9% of participants owned smartphones and their median self-reported digital literacy index was 3. Factors associated with phone ownership included younger age and higher education levels. Factors associated with increased propensity to enroll in a DLP included being non-Chinese (ethnic minority), more educated, have a smartphone and better self-reported digital literacy. Socio-demographic factors found to be associated with smartphone ownership and better digital literacy mostly corroborate with existing literature (17, 18).

These factors found to increase one's propensity to enroll in a DLP highlight the fact that those who are at even greater risk of being left behind by the digital divide (e.g., less educated, lacking a smartphone, ethnic minority) remain under-served. While looking to ICT to mitigate these negative consequences (Newman & Zainal, 2020), communities must be cognizant of older adults who are unlikely to realize the promise of ICT due to relatively low smartphone ownership and poor digital literacy. Further efforts to engage and reach out to this group to address their unmet needs are necessary to reduce disparities.

This study elucidated that social digital literacy had an indirect positive effect on both well-being and quality of life, mediated by social connectedness and perceived loneliness, while instrumental digital literacy had an opposite effect. The results from the serial mediation model demonstrated how better social digital literacy leads to more robust connectedness, thereby less loneliness, consequently leading to a positive impact on wellbeing and quality of life to a smaller extent. However, there is a negative relationship between instrumental literacy (the use of applications to perform daily but non-social and non-entertainment related tasks) and wellbeing and quality of life. One plausible explanation for this observation is that social isolation and poorer quality of life necessitate greater independence. They may foster greater proficiency in instrumental and reassurance-use functions in an increasingly digital world. This postulation of a complex relationship between social isolation and independence requires further testing using longitudinal research. Furthermore, not all aspects of digital literacy are equally important to wellbeing. Reassurance literacy and pastime literacy were found not to be related to wellbeing and quality of life.

The current set of findings is not free from several methodological limitations. Firstly, given the convenient sampling of the current samples, generalizability of the findings to other samples of the same population of older adults of low SES will have to be tested with independent studies. Secondly, while it is tempting to interpret the associations found in the mediation model as inherently causal; readers should note that the data in this study was collected from a cross-sectional study, therefore longitudinal studies need to be conducted to establish the temporal causal relationship.

Looking forward, this study highlights the key factors to partake in a DLP and suggests that DLP focusing on increasing social digital literacy may potentially reduce social isolation, loneliness and improve wellbeing and quality of life. Such an impact should

continue to motivate efforts to close the digital divide; particularly to try to reach out to the few who are most at risk but remain most difficult to reach.

Supporting Information

S1 Appendix. Supplementary Tables.

Acknowledgments

The authors wish to thank Ming Hao Wee, Wee Teck Loh from NTUC Health Co-operative Ltd; Montfort Care; Infocomm Media Development Authority; Singapore General Hospital Community Nursing and Hospital-to-Home Program; Elizabeth Puay Ting Pang, Jamaica Pei Ying Tan from Singapore General Hospital Population Health and Integrated Care Office. This study would not be possible without the support of these individuals and organizations.

References

1. Wu B. Social isolation and loneliness among older adults in the context of COVID-19: a global challenge. Global Health Research and Policy. 2020;5(1):27.
2. Gariépy G, Honkaniemi H, Quesnel-Vallée A. Social support and protection from depression: systematic review of current findings in Western countries. Br J Psychiatry. 2016;209(4):284-93.
3. Ge L, Yap CW, Ong R, Heng BH. Social isolation, loneliness and their relationships with depressive symptoms: A population-based study. PLOS ONE. 2017;12(8):e0182145.
4. Santini ZI, Jose PE, York Cornwell E, Koyanagi A, Nielsen L, Hinrichsen C, et al. Social disconnectedness, perceived isolation, and symptoms of depression and anxiety among older Americans (NSHAP): a longitudinal mediation analysis. The Lancet Public Health. 2020;5(1):e62-e70.
5. Chen Y, Feeley TH. Social support, social strain, loneliness, and well-being among older adults:An analysis of the Health and Retirement Study*. Journal of Social and Personal Relationships. 2014;31(2):141-61.
6. Holt-Lunstad J, Steptoe A. Social isolation: An underappreciated determinant of physical health. Current Opinion in Psychology. 2022;43:232-7.
7. Holt-Lunstad J, Smith TB, Layton JB. Social Relationships and Mortality Risk: A Meta-analytic Review. PLOS Medicine. 2010;7(7):e1000316.
8. Hawkley LC, Cacioppo JT. Loneliness matters: a theoretical and empirical review of consequences and mechanisms. Ann Behav Med. 2010;40(2):218-27.
9. Flaxman S, Mishra S, Gandy A, Unwin HJT, Mellan TA, Coupland H, et al. Estimating the effects of non-pharmaceutical interventions on COVID-19 in Europe. Nature. 2020;584(7820):257-61.
10. Pan A, Liu L, Wang C, Guo H, Hao X, Wang Q, et al. Association of Public Health Interventions With the Epidemiology of the COVID-19 Outbreak in Wuhan, China. JAMA. 2020;323(19):1915-23.
11. Clair R, Gordon M, Kroon M, Reilly C. The effects of social isolation on well-being and life satisfaction during pandemic. Humanities and Social Sciences Communications. 2021;8(1):28.
12. Smart Nation Singapore. Transforming Singapore Through Technology 2021 [Available from: https://www.smartnation.gov.sg/about-smart-nation/transforming-singapore.
13. Newman MG, Zainal NH. The value of maintaining social connections for mental health in older people. The Lancet Public Health. 2020;5(1):e12-e3.
14. Chopik WJ. The Benefits of Social Technology Use Among Older Adults Are Mediated by Reduced Loneliness. Cyberpsychol Behav Soc Netw. 2016;19(9):551-6.
15. Millard A, Baldassar L, Wilding R. The significance of digital citizenship in the well-being of older migrants. Public Health. 2018;158:144-8.
16. Llorente-Barroso C, Kolotouchkina O, Mañas-Viniegra L. The Enabling Role of ICT to Mitigate the Negative Effects of Emotional and Social Loneliness of the Elderly during

COVID-19 Pandemic. International Journal of Environmental Research and Public Health. 2021;18(8):3923.
17. Fang ML, Canham SL, Battersby L, Sixsmith J, Wada M, Sixsmith A. Exploring Privilege in the Digital Divide: Implications for Theory, Policy, and Practice. The Gerontologist. 2018;59(1):e1-e15.
18. Ang S, Lim E, Malhotra R. Health-Related Difficulty in Internet Use Among Older Adults: Correlates and Mediation of Its Association With Quality of Life Through Social Support Networks. The Gerontologist. 2020;61(5):693-702.
19. Choi NG, Dinitto DM. Internet use among older adults: association with health needs, psychological capital, and social capital. J Med Internet Res. 2013;15(5):e97-e.
20. Steptoe A, Di Gessa G. Mental health and social interactions of older people with physical disabilities in England during the COVID-19 pandemic: a longitudinal cohort study. The Lancet Public Health. 2021;6(6):e365-e73.
21. IMDA. Seniors Go Digital 2021 [Available from: https://www.imda.gov.sg/en/seniorsgodigital.
22. Schreurs K, Quan-Haase A, Martin K. Problematizing the Digital Literacy Paradox in the Context of Older Adults' ICT Use: Aging, Media Discourse, and Self-Determination. Canadian Journal of Communication. 2017;42(2):359-77.
23. Ministry of Communications and Information. Digital Readiness Blueprint 2021 [Available from: https://www.mci.gov.sg/en/portfolios/digital-readiness/digital-readiness-blueprint.
24. Dimmick JW, Sikand J, Patterson SJ. The Gratifications of the Household Telephone: Sociability, Instrumentality, and Reassurance. Communication Research. 1994;21(5):643-63.
25. Wang Y, Matz-Costa C, Miller J, Carr DC, Kohlbacher F. Uses and Gratifications Sought From Mobile Phones and Loneliness Among Japanese Midlife and Older Adults: A Mediation Analysis. Innov Aging. 2018;2(3):igy027-igy.
26. Lubben J, Blozik E, Gillmann G, Iliffe S, von Renteln Kruse W, Beck JC, et al. Performance of an Abbreviated Version of the Lubben Social Network Scale Among Three European Community-Dwelling Older Adult Populations. The Gerontologist. 2006;46(4):503-13.
27. Russell D, Peplau LA, Cutrona CE. The revised UCLA Loneliness Scale: concurrent and discriminant validity evidence. Journal of personality and social psychology. 1980;39(3):472-80.
28. Benson T, Sladen J, Liles A, Potts HWW. Personal Wellbeing Score (PWS)—a short version of ONS4: development and validation in social prescribing. BMJ Open Quality. 2019;8(2):e000394.
29. Rabin R, Gudex C, Selai C, Herdman M. From translation to version management: a history and review of methods for the cultural adaptation of the EuroQol five-dimensional questionnaire. Value Health. 2014;17(1):70-6.
30. Luo N, Wang P, Thumboo J, Lim YW, Vrijhoef HJ. Valuation of EQ-5D-3L health states in Singapore: modeling of time trade-off values for 80 empirically observed health states. Pharmacoeconomics. 2014;32(5):495-507.
31. Vyas S, Kumaranayake L. Constructing socio-economic status indices: how to use principal components analysis. Health Policy Plan. 2006;21(6):459-68.
32. Morton F, Nijjar JS. eq5d. 0.10.0 ed2021.
33. Hu L-t, Bentler PM. Cutoff criteria for fit indexes in covariance structure analysis: Conventional criteria versus new alternatives. Structural Equation Modeling. 1999;6(1):1-55.

34. Department of Statistics Singapore. Population Trends 2019. 2019.

Figure

Click here to access/download;Figure;Fig1.tif

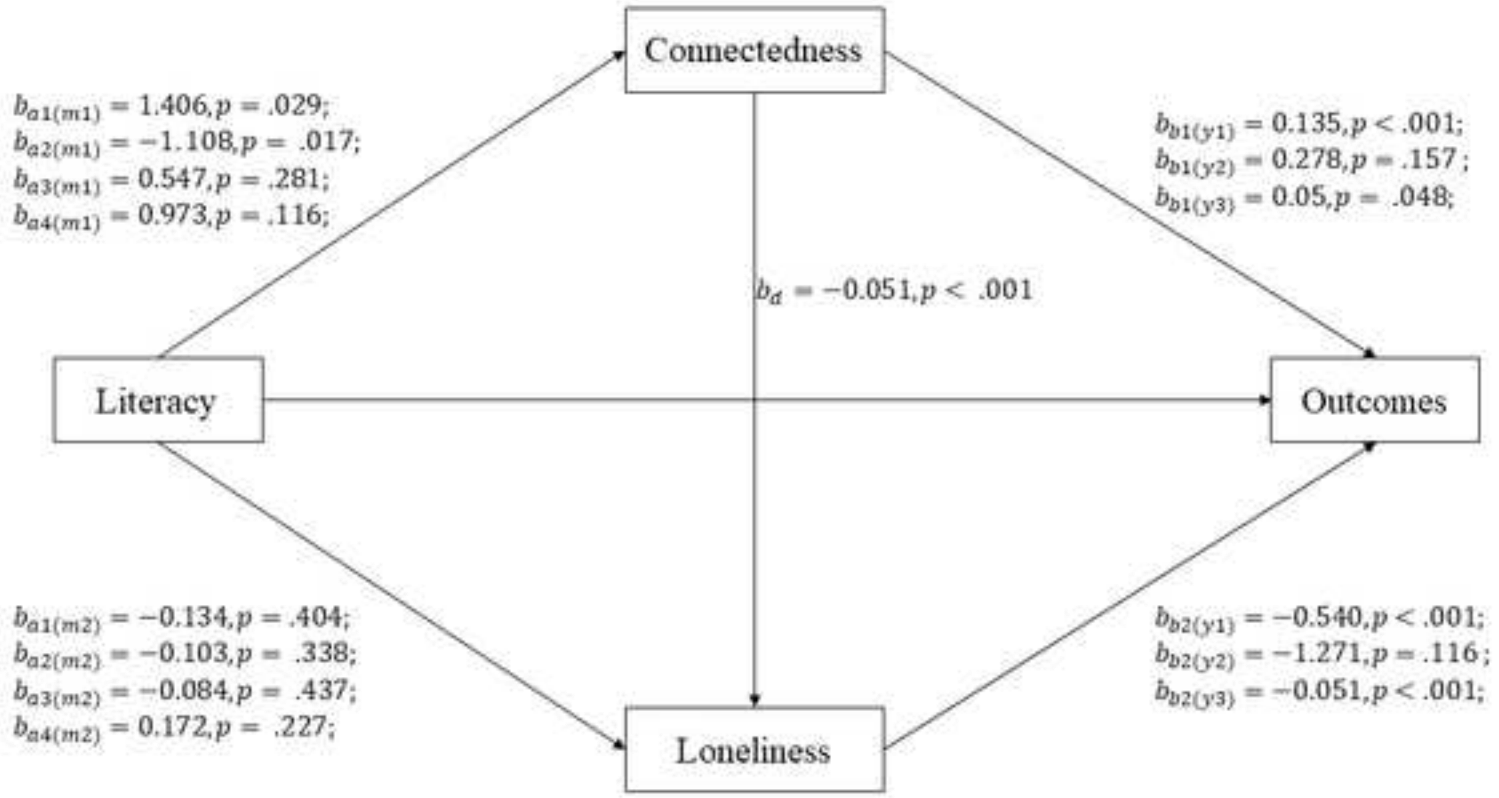

Click here to access/download
Supporting Information
S1 Appendix.docx
