# Supplementary material for: Smartphone ownership, digital literacy, and the mediating role of social connectedness and loneliness in improving the wellbeing of community-dwelling older adults of low socio-economic status in Singapore
Source: PLoS One. 2023 Aug 30;18(8):e0290557. doi: 10.1371/journal.pone.0290557 (PMC10468072; doi:10.1371/journal.pone.0290557)
Supplement: Attachment — Submitted filename: Response to Reviewers.docx [file pone.0290557.s003.docx]

**Manuscript** PONE-D-23-04295**: Title** Smartphone Ownership, Digital Literacy, and the Mediating Role of Social Connectedness and Loneliness in Improving the Wellbeing of Community-Dwelling Older Adults of Low Socio-Economic Status in Singapore

We thank the editor and reviewers for taking time out from their busy schedules to provide a constructive critique that has helped improve our manuscript. We have provided responses to each comment below. We have specified the page and line numbers for the changes and this can be corroborated to the ‘*clean manuscript’* document*.* Alternatively, the changes can also be tracked in the ‘*revised manuscript with changes*’ document.

**IN RESPONSE TO THE REVIEWER’S COMMENTS:**

**REVIEWER 1:**

**A1. COMMENT TO AUTHOR:** This research is very well done. My comments are seeking for consistency between the results (text) and the values given in Figure 1. Please see comment bubbles in the attachment. Please clarify the values, if needed.

**A1. RESPONSE:**

Thank you for your feedback. We have inspected Fig 1 and noticed that the figure had incomplete data. We have since updated Fig 1, resolving the inconsistency between the main text and the figure. Please see a reproduction of the updated Fig 1 below.

**REVIEWER 2:**

**B1. COMMENT TO AUTHOR:**

The study seeks to explore the connection between digital literacy and various factors such as social connectedness, loneliness, wellbeing, and quality of life (QoL) through mediation analysis. Extensive research has demonstrated the significant influence of social isolation and loneliness on the overall health, mental well-being, and longevity of older individuals, as well as their quality of life. To address these issues, numerous interventions, both face-to-face and digital, have been developed to mitigate social isolation and loneliness among older adults. Given this context, the current manuscript is intriguing and appears to contribute to our existing knowledge by examining the role of digital literacy as a potential mediator in enhancing the well-being of vulnerable elderly individuals.

However, I have the following concerns that needs to be addressed for better clarity and further improvement:

Title: Title needs to be clear, specific and reflect the content and context of the manuscript. It should clearly delineate exposure, outcome, and mediator variables. I presume it is “social connectedness” not “connectedness”

**B1. RESPONSE:**

Thank you for your feedback. We have modified the title and changed from “connectedness” to “social connectedness”. The modified full title would be “Smartphone Ownership, Digital Literacy, and the Mediating Role of Social Connectedness and Loneliness in Improving the Wellbeing of Community-Dwelling Older Adults of Low Socio-Economic Status in Singapore”.

**B2. COMMENT TO AUTHOR:**

Abstract appears too brief to provide a clear picture for the readers. The abstract can be expanded to better reflect the manuscript's content and context. Information on analytic approach used for mediation analyses and results (including point estimates and uncertainty estimates) should be provided.

**B2. RESPONSE:**

Thank you for your feedback. We have modified our abstract to provide more details on the analysis including point estimates and the confidence interval associated with the key findings that we wished to highlight. The updated abstract can be found in *Page 3-4, Line 39 to 72*. The updated abstract is also produced below for your reference with the changes bolded and italicized.

***Introduction***

During the COVID-19 pandemic, safe-distancing measures resulted in many community-dwelling older adults being socially isolated and lonely, with its attending negative impact on wellbeing and quality of life. While digital technology may have mitigated this, older adults of low socioeconomic status (SES) are more likely to be digitally excluded and hence susceptible to the adverse effects of social isolation and loneliness. ***This study aims to understand the factors that affect digital literacy, smartphone ownership, and willingness to participate in a digital literacy program (DLP), and to test the hypothesized relations between digital literacy, social connectedness, loneliness, wellbeing, and quality of life amongst community dwelling older adults of low SES.***

***Materials and Methods***

***A questionnaire assessing digital literacy, social connectedness, wellbeing and quality of life was administered. Socio-demographic variables, pre-existing internet-enabled, and willingness to participate in a home-based DLP was also collected.*** Logistic regression was used to identify demographic factors associated with digital literacy, smartphone ownership, and willingness to enrol in a DLP. Serial mediation analysis was also performed using a structural equation model framework.

***Results***

A total of 302 participants were recruited. Female gender, older age, lower education levels were associated with lower digital literacy. Those who owned a smartphone tended to be younger and better educated. Older adults who were better educated, of Chinese descent (the ethnic majority in Singapore), and who had higher digital literacy, were most willing to enroll in the digital literacy education program. Social-use digital literacy had a positive indirect effect on well-being ($\hat{b}=0.037, 95\% CI=[0.007, 0.101]$) and Quality of life ($\hat{b}=0.004, 95\% CI=[0.001, 0.010]$), mediated by social connectedness and loneliness. In contrast, instrumental-use digital literacy had a negative indirect effect on well-being ($\hat{b}=-0.030, 95\% CI=[-0.080, -0.008]$) and Quality of life ($\hat{b}=-0.003, 95\% CI=[-0.008, -0.001]$), mediated by social connectedness and loneliness.

***Discussion***

***The results suggest there are demographic barriers to participation in DLPs and highlight the benefit of focusing on enhancing social-use digital literacy.*** Further study is needed to evaluate how well specific interventions to improve social-use digital literacy help to reduce social isolation and loneliness, and ultimately improve wellbeing and quality of life.

**B3. COMMENT TO AUTHOR:**

Introduction: The description of social connectedness in the opening statement does not seem valid (?? Absence).

For better clarity and flow, the introduction needs to provide adequate information on the vulnerability of elderly for social isolation and loneliness and its consequences before describing Digital Transformation Master Plan.

The introduction can include a brief description of digital literacy in a line or two.

As “propensity” has statistical interpretation; the authors can use appropriate alternate term for “propensity”.

The supporting evidence for why the mediators might affect the outcomes (wellbeing and quality of life of older adults) needs to be strengthened.

Overall, the introduction provides a good description of the study background and theoretical rationale for investigating the mechanisms of interest. The theory that underpins the proposed mechanisms of interest and why the exposure or intervention is expected to affect the proposed mediator (action theory), and why the mediator is expected to affect the outcome (conceptual theory) is also stated explicitly.

**B3. RESPONSE:**

Thank you for your feedback. The introduction has been modified to highlight the (1) risk of social isolation and loneliness in older adults and its consequence *(Page 5, Line 74-83),* (2) concept of digital literacy *(Page 5, Line 86-87)* and (3) supporting evidence on the relationship between the mediators and outcomes. *(Page 5, Line 81-83 and Page 7, Line 122-128).*

In brief, we first introduced the impact of social connectedness and loneliness on health and non-health outcomes in the first paragraph of the introduction. Then we built a case for the positive effect of ICT on improving social connectedness and loneliness, and the need to enhance ICT use via increasing digital literacy. Then we provide a summary of the literature on the relationship between digital literacy, social connectedness, loneliness, and wellbeing and quality of life, and proposed our hypothesis on the serial mediation mechanism based on the literature.

Additionally, we have also replaced the word “propensity” in the paper with “willingness” to reflect the intention to participate in the digital literacy programme as suggested.

**B4(i). COMMENT TO AUTHOR:**

Materials & Methods: The statement “The study was reported following the Strengthening the Reporting of Observational Studies in Epidemiology (STROBE) guidelines for cross-sectional studies” appears irrelevant. Methods should focus on how it was conducted not how it was reported.

**B4(i). RESPONSE:**

Thank you for your feedback. We have opted to include the statement regarding reporting in accordance to Strengthening the Reporting of Observational Studies in Epidemiology (STROBE) guidelines. However, we acknowledge that the methods section should focus on how it was conducted and not how it was reported. Hence, we have re-organized the methods segment to prioritize the content on how the study was conducted by moving the STROBE from the top of the Materials and Methods section to the end of the section *(Page 11, Line 226-227)*.

**B4(ii). COMMENT TO AUTHOR:**

The authors can mention the rationale for including those aged >55 years as the definition of elderly varies across the countries.

Exclusion criteria needs to be mentioned explicitly. For example, one is not sure about participants who were already enrolled and participated in DLP.

**B4(ii). RESPONSE:**

Thank you for your feedback. We have included an elaboration on the reasons in using 55 years old as a low threshold for being an older adult in the study, as well as the exclusion criteria. The use of 55 years old is in-line with the convention of local research projects using older adults as samples, such as Lee et al.’s (2022) longitudinal study on ageing in Singapore, which we have included as a citation in the manuscript.

The changes can be found on *Page 8, Line 143-144* of the manuscript. We would like to highlight that as this study aimed to understand the factors that affect willingness to partake in DLP, we included participants willing to partake in this study regardless of enrolment status in DLP.

**B4(iii). COMMENT TO AUTHOR:**

Sampling strategy needs expansion for better clarity. It is possible that different strategies “phone calls and door-to-door outreach” would have introduce selection bias by deviating from randomness.

Reason for differential consent procedure for those who participate and those who do not participate in DLP and its ethical validity.

Possible measurement bias due to different data collection strategies should be mentioned in discussion section.

**B4(iii). RESPONSE:**

Thank you for your feedback. As a response to the sampling strategy, we used the convenience sampling method to recruit our participants. Our approach consisted of engaging with individuals, who have been identified by local community eldercare and welfare service providers, to be aged >55 years and belonging to lower SES (residing in public rental housing or receiving welfare payments from the Public Assistance Scheme). To increase participation rates, the team first started off by reaching out to participants via phone calls. If the individual did not pick up the call despite three attempts made at different days and times, at least two physical visits would be conducted (once on a weekday and once on a weekend) to recruit the individual. Failing which, the individual will be considered as not contactable. *(Page 8, Line 146-152)*

As such, we acknowledge the sample’s potential lack of representation of the population as an inherent limitation to our limitation, and we have discussed this limitation in the Discussion section *(Page 26, Line 397-400)*.

The differentiated consent taking was approved by the SingHealth Centralized Institutional Review Board (2020/2722). It was deemed that verbal consent would suffice for participants who agreed to partake in the research but were not willing to partake in the DLP because the anonymized data collected posed minimal risk to them.

Responding to the comment on the data collection strategy, the data was collected using standardized procedures either via face-to-face or via phone. Given the procedures were standardized with the only difference in the medium of communication, we do not expect systematic bias that would impact the inference of the results.

We have added additional information to describe the data collection standardized procedure and ethical approval at the end of the Participants and Procedure subsection of the Materials and Methods section *(Page 8, Line 160-161).*

**B4(iv). COMMENT TO AUTHOR:**

Languages used for questionnaire administration needs to be mentioned.

It should be “questionnaire administration” not “survey administration”

**B4(iv). RESPONSE:**

Thank you for your feedback. Questionnaire was administered in English or Mandarin, in line with the exclusion criteria on the requirement on spoken language. We have changed “survey administration” to “questionnaire administration” *(page 8, Line 157)* and included the language the questionnaire was administered in *(page 8, Line 156-158)*

**B4(v). COMMENT TO AUTHOR:**

Though the authors have used the term “digital literacy”, it was smartphone literacy that was specifically addressed. This needs to be reflected in the title and discussion.

**B4(v). RESPONSE:**

Thank you for your feedback. We have opted to keep the term “digital literacy” instead of changing it to “smartphone literacy” as the focus of the program was to improve participants’ proficiency in activities of digital literacy such as using the internet or to access information or service. This could be done through any medium such as the phone, computer or tablets as preferred by the participants. This is also in-line with the local government’s initiative in increasing digital literacy among older adults via the provision of subsidized smartphones and data plans. The internet is accessed via a smartphone by most of the population in Singapore.

We hope this response is acceptable to the reviewer.

**B4(vi). COMMENT TO AUTHOR:**

For all the study instruments used, scoring pattern, maximum and minimum possible score range and basic psychometric proprieties should be mentioned.

**B4(vi). RESPONSE:**

Thank you for your feedback. The theoretical minimum and maximum score of each of the measurement can be found in Table S2 and their reliability are reported in the results section (*page 19, Line 301-303).* The scoring method is described in the Instruments section *(page 9, Line 192-196)*.

**B4(vii). COMMENT TO AUTHOR:**

From the description, it was not clear how information on socio-demographic variables, smartphone ownership, as well as participants’ propensity to enroll in a DLP was collected.

**B4(vii). RESPONSE:**

We have included how information on socio-demographic variables and smartphone ownership were collected *(Page 8, Line 163-164).* Inclination to join a DLP was determined by self-reported willingness to join the homebased DLP offered by the study. The DLP was a collaboration between TriGen (a charity), Singapore General Hospital, Infocomm Media Development Authority (key government institution in Singapore managing information communications and media), and senior activity centers as described in the paper *(Page 6, Line 113-118).*

**B5(i). COMMENT TO AUTHOR:**

Data analysis: The reference for the formula used to derive the minimum sample size estimation based on Monte Carlo simulations should be provided.

The authors should reconfirm whether it was bivariate or Univariable analysis that was performed to find the associations.

**B5(i). RESPONSE:**

Thank you for your feedback. The Monte Carlo simulation methodology was based on Muthen and Muthen’s (2019) suggestions and procedure, which we have included as a reference in the main text *(Page 9, Line 181-184).* We used the term “univariable” to describe the process of relating each predictor to the outcome independently. We have added a footnote in the main text to elaborate on our terminology to avoid confusion. *(Page 18, Line 291-292).*

**B5(ii). COMMENT TO AUTHOR:**

Specific assumptions about the causal model (effect modification, positivity, and consistency) , if any can be elaborated.

Also, assumptions on assumptions of multivariate normality and linearity (if any), and post-hoc modifications (if any) can be mentioned. The authors can mention the approach (if any) that was used to explore violation of assumptions with regard to residual confounding, direction of causal relationships and absence of common causes of multiple mediators. This will ensure more robustness and reliability of the analysis.

**B5(ii). RESPONSE:**

Thank you for your feedback. We believe the causal inference assumptions of positivity and consistency, which both relate to treatment exposure, are not relevant in our paper because the participants were not subjected to any interventions (for participants who agreed to participate in the Digital Literacy Programme, the data relevant to this study was collected prior to them enrolling in the programme)Statistical assumption of multivariate normality was also circumvented by the choice of using bootstrapped standard error and confidence interval rather than assuming a normally distributed test statistic.

We conceptualized the serial mediation model based on the literature and the model goodness-of-fit indices suggested that our hypothesized model was a good fit to the data, hence we concluded that the hypothesised causal relationships were a good reflection of the data. For these reasons, no post-hoc modification were conducted.

The major limitation of establishing the direction of causal relationship using cross-sectional data was elaborated in the Discussion section *(Page 26-27, Line 400-403).*

**B5(iii). COMMENT TO AUTHOR:**

Authors can mention whether there was clustering due to their sampling strategy; if there was clustering, should describe how clustering was accounted for with regard to within- and between-cluster heterogeneity, and possible effects by participation in DLP programme for the estimation of direct and indirect effects.

**B5(iii). RESPONSE:**

Thank you for your feedback. We did not expect clustering due to our sampling of the samples from the population of interest as the neighbourhoods were homogenous (residence of public rental housing or on welfare payments from the Southern region of Singapore). Furthermore, the set of data used in this paper was collected before the participation of the DLP and hence, there is no expected confounding effect of programme participation as an intervention.

**B6(i). COMMENT TO AUTHOR:**

Results : The authors should mention the difference between mobile and smartphone and the relevance of describing mobile phone with regard to the stated objectives and included key concepts.

**B6(i). RESPONSE:**

Thank you for your feedback. We have added a footnote in Table 1 *(page 12-13)* to describe the difference between mobile phone and smartphone. The footnote is provided here for your reference – “*Smartphone is a subset of mobile phone. Mobile phones refer to any types of phones that are not connected to a physical landline, therefore consists of both featurephone and smartphone.”*

**B6(ii). COMMENT TO AUTHOR:**

As table 3 reflects the primary objective, the logistic regression model should include all the variables and provide both adjusted and unadjusted odds ratio for all the variables.

**B6(ii). RESPONSE:**

Thank you for your feedback. We aimed to create a parsimonious logistic regression model for interpretability, hence we had only included a subset of variables of interests (i.e., age, gender, race, education level, housing type, apartment size, prior smartphone possession, digital literacy) in the logistic regression, as informed by the feature selection process using the non-parametric chi-square tests and Mann-Whitney U-Tests which do not produce the odds ratio model parameter.

**B6(iii). COMMENT TO AUTHOR:**

The authors should reorganise the mediation results as per the stated hypothesis for better clarity.

**B6(iii). RESPONSE:**

Thank you for your feedback. As per suggestion, we have reorganized the results of the mediation model to place a stronger emphasis on the hypotheses on the serial mediation effect in the main text by removing results that are not related to the serial mediations. Table 4 has been shortened to only contain the key parameters of interest. The presentation of Figure 1 remained unchanged to allow the readers to evaluate our findings and conclusions.

**B7(i). COMMENT TO AUTHOR:**

Discussion: Overall, the discussion appears weak, terse and needs detailed elaboration by comparing with existing evidence for the stated objectives and hypothesis.

The discussion can highlight how the result findings concord with the stated objectives and hypothesis.

**B7(i). RESPONSE:**

Thank you for your feedback. We have modified the discussion on the findings with relations to the literature and hypothesis.

The first study objective was to understand the factors that affect digital literacy, smartphone ownership, and the willingness for DLP participation. We demonstrated that female, older age, and lower education levels were associated with lower digital literacy. Factors associated with smartphone ownership included younger age and higher education levels. In addition, factors associated with increased willingness to enrol in a DLP included being non-Chinese (ethnic minority), more educated, have a smartphone and better self-reported digital literacy. This corroborates with the existing literature that shows a correlation between sociodemographic factors and digital literacy. This can be found between *Page 25, Line 354-361.*

The second objective aims to test the relationship between digital literacy, social connectedness, loneliness, and wellbeing and quality of life. Our serial mediation model demonstrated that enhanced social digital literacy resulted in more robust connectedness, thereby less loneliness, consequently having a positive impact on wellbeing and quality of life to a smaller extent. In contrast, there was a negative relationship observed between instrumental digital literacy (the use of applications to perform daily but non-social and non-entertainment related tasks) and wellbeing and quality of life. One plausible explanation for this observation is that social isolation and poorer quality of life necessitates greater independence, fostering greater proficiency in instrumental functions in an increasingly digital world. This can be found between *Page 25-26, Line 370-386.*

**B7(ii). COMMENT TO AUTHOR:**

Authors can state any limitations regarding unmeasured confounding, measurement error, model misspecification, selection bias, and missing data.

The authors can bring out the chain mediating effect and its implications in their discussion section.

**B7(ii). RESPONSE:**

Thank you for your feedback. We have addressed several major methodological limitations that exist within the present study. Firstly, given the convenient sampling of the current samples, generalizability of the findings to other samples of the same population of older adults of low SES will have to be tested with independent studies. Secondly, given that the data in this study was collected from a cross-sectional study, longitudinal studies need to be conducted to establish the temporal causal relationship. This can be found between *Page 26-27, Line 397-403.*

We do not consider measurement error as a key limitation in our study as the measurements were largely reliable based on the Cronbach’s alpha internal consistency estimates. The mediation model goodness-of-fit indices also showed a satisfactory data-model fit, which served as evidence of a general absence of significant model misspecification. Missing data in the mediation model was also handled with the full information maximum likelihood method along with the several auxiliary variables to account for the presence of missing data, which should theoretically provide us with estimates with lower bias and uncertainty.

The relationship between digital literacy, social connectedness, loneliness, wellbeing and quality of life has been evaluated in a piecemeal manner in the literature. The results of the serial mediation model suggested a potential unified causal framework of digital literacy and wellbeing and quality of life through social connectedness and loneliness. Furthermore, the results showed that not all types of digital literacy were related to wellbeing and quality of life. Thus, taken all together, the results could be used to inform the design of digital literacy programme. These implications of the results were contained between *Page 25-26, Line 370-396.*

**B7(iii). COMMENT TO AUTHOR:**

Authors should add a para on implications of the study findings.

Further, the authors should revisit the results section and shift that information which were not discussed into the supplementary appendix.

**B7(iii). RESPONSE:**

Thank you for your feedback. We have modified the discussion segment to include the implications of the study findings. Findings from this study have implications on the considerations of implementing programs to improve digital literacy in older adults. Lower socioeconomic status, lower education levels and older age are barriers to DLP uptake and participation. Extra effort is required to reach these hard-to-reach group. We highlighted the need to be cognizant that not all aspects of digital literacy are equally important to wellbeing, with social digital literacy being associated with an increased social connectedness and reduced loneliness, and consequently, improvement in wellbeing and QoL. Policy planners and organizers of DLP should consider dedicating more resources to certain aspects of digital literacy. This is delineated from *Page 26, Line 387-396.*

Table 4 in the result section has been shortened to only contain the key parameters related to serial mediation. We shifted the original Table 4 to the Supplementary Materials and labelled it as Table S5.

Once again, thank you to both reviewers’ for the feedback.

Kind regards,

Dr Kennedy Ng

On behalf of all authors
