# Supplementary material for: Smartphone ownership, digital literacy, and the mediating role of social connectedness and loneliness in improving the wellbeing of community-dwelling older adults of low socio-economic status in Singapore
Source: PLoS One. 2023 Aug 30;18(8):e0290557. doi: 10.1371/journal.pone.0290557 (PMC10468072; doi:10.1371/journal.pone.0290557)
Supplement: Attachment — Submitted filename: Response to Reviewers.docx [file pone.0290557.s004.docx]

**Manuscript** PONE-D-23-04295**: Title** Smartphone Ownership, Digital Literacy, and the Mediating Role of Social Connectedness and Loneliness in Improving the Wellbeing of Community-Dwelling Older Adults of Low Socio-Economic Status in Singapore

In response to the reviewer’s comments dated 3 July 2023:

**A1. COMMENT TO AUTHOR:** The authors response that they did not expect clustering due to our sampling requires more objective evidence. One can find the clustering at the institution level from where the participants were sampled. In this context, the authors need to provide objective evidence that there is no clustering in their data. Consequently, any finding related to clustering should be discussed in the manuscript.

**A1. RESPONSE:**

Thank you for your thoughtful comments regarding potential clustering due the sampling. We acknowledge the need for objective evidence to support our expectation on the absence of significant clustering in our data. We considered the recruitment sites (including eldercare centers and welfare service providers) and residence neighbourhood as potential clustering units. Unfortunately, these data were partially collected for administrative purposes and were not included in Institutional Review Board (IRB) application for ethical approval. Hence, we do not have sufficient data on these clustering units to perform the within- and between-cluster variance decomposition required to establish the intra-class correlations to evaluate clustering. Therefore, we decided to include the untested assumption of the absence of clustering as a limitation of this study and discuss its potential impact on the statistical conclusion in the discussion section.

Please see line **402-405 on pages 27-28 and the footnote on page 28** for the newly included text.

**A2. COMMENT TO AUTHOR:** The authors aim to create parsimonious logistic regression model need strong justification. With the exclusion of age and gender which are considered to be universal confounders, the current model falls short to provide robust and valid estimates (The current model wrongly rejects potentially important variables when the relationship between an outcome and a risk factor is confounded and through exclusion when this confounder is not properly controlled). There is possibility that inclusion of age and gender might turn some of the significant variables to non-significant ones.

Rather than parsimonious, the logistic regression model should be robust and more valid. The most unpleasant side effect of variable selection is its impact on inference about true values of regression coefficients by means of tests and confidence intervals. Further, using a threshold P-value <0.05 for variable selection often led to important adjustment variables being dropped from a model due to stochastic variability. Hence, if the authors still want to proceed with variable selection method, they should use the recommended less stringent threshold (P-value <0.25) for variable selection and provide evidence on selection stability and model uncertainty through sensitivity analyses.

**A2. RESPONSE:**

Thank you for your insightful feedback about the robustness of the regression model. We acknowledge the valid concerns you have raised about excluding variables based on stochastic estimates of relationships based on univariable analyses. Hence, we have opted to build a more comprehensive model that includes age and gender and updated **Table 3** accordingly **(pages 17 to 19)**. The apartment size variable continues to be excluded from the model, not based on stochastic estimates, but because of the relationship between ownership status and apartment size that is idiosyncratic to Singapore’s public housing system – that one-room apartments in public housing blocks are necessarily rented from the Housing Development Board itself, while two-room and larger apartments are purposed for owner-occupants. This explanation has been furnished as a footnote to **Table 3** on **Page 18** in the manuscript.
